# Comparison of serum and plasma as a source of blood extracellular vesicles: Increased levels of platelet-derived particles in serum extracellular vesicle fractions alter content profiles from plasma extracellular vesicle fractions

**DOI:** 10.1371/journal.pone.0270634

**Published:** 2022-06-24

**Authors:** Xiaoman Zhang, Toshihide Takeuchi, Akiko Takeda, Hideki Mochizuki, Yoshitaka Nagai

**Affiliations:** 1 Department of Neurotherapeutics, Osaka University Graduate School of Medicine, Suita, Osaka, Japan; 2 Department of Neurology, Kindai University Faculty of Medicine, Osaka-Sayama, Osaka, Japan; 3 Life Science Research Institute, Kindai University, Osaka-Sayama, Osaka, Japan; 4 PRESTO, Japan Science and Technology Agency (JST), Osaka, Japan; 5 Department of Neurology, Osaka University Graduate School of Medicine, Suita, Osaka, Japan; National Institute of Child Health and Human Development, UNITED STATES

## Abstract

Extracellular vesicles (EVs) have attracted much attention as potential diagnostic biomarkers for human diseases. Although both plasma and serum are utilized as a source of blood EVs, it remains unclear whether, how and to what extent the choice of plasma and serum affects the experimental results. To address this issue, in this study, we performed comprehensive characterization of EV fractions derived from plasma and serum, and investigated the differences between these blood EVs. We demonstrated by nanoparticle tracking analysis that EV fractions derived from serum contain more particles than those from plasma of mice. Proteomic analysis demonstrated that platelet-associated proteins are selectively enriched in serum EV fractions from both mice and humans. A literature review of proteomic data of human blood EVs reported by other groups further confirmed that selective enrichment of platelet-associated proteins is commonly observed in serum EVs, and confers different proteome profiles to plasma EVs. Our data provide experimental evidence that EV fractions derived from serum generally contain additional EVs that are released from platelets, which may qualitatively and quantitatively alter EV profiles when using serum as a source of blood EVs.

## Introduction

Extracellular vesicles (EVs) are a group of membrane particles that are released from cells, including exosomes, microvesicles and other vesicular components [1, 2]. They are abundantly present in extracellular fluids, such as circulating blood, cerebrospinal fluid, and urine, and carry a variety of proteins, nucleic acids, lipids, and metabolites as their cargoes. Cell-to-cell transmission is a characteristic of EVs, which mediates the intercellular transfer of their cargoes from donor cells to recipients, enabling non-cell autonomous control of the immune response, gene transcription, and other cellular functions at the multicellular organismal level [3–5].

Because the cargoes of EVs are likely to vary depending on not only the cell type but also the state of the cells (particularly under stressed or disease conditions), EVs have attracted much attention as potential diagnostic biomarkers for human diseases [6, 7]. Among extracellular fluids, EVs derived from blood are one of the most promising sources for biomarker development, owing to the ease of sampling blood with minimal invasion. To date, many studies analyzing biomarker candidates from blood-derived EVs have been reported, for several types of cancers [8–10], immune diseases [11, 12], and neurodegenerative diseases, such as Alzheimer’s disease and Parkinson’s disease [13, 14]. However, the development of reliable EV-based biomarkers applicable for clinical use remains challenging, as even for the same disease, the results of EV analyses of patients’ blood are often inconsistent among studies. A major reason for these inconsistent results is the difference in experimental conditions, because blood EV profiles are likely to be affected by various experimental factors, such as the sample source, EV isolation method, and the choice of plasma or serum.

Although plasma and serum, which are the two major types of blood supernatant, are used as a source of blood EVs, it remains unclear whether, how, and to what extent the choice of plasma and serum actually affects the results and interpretation of blood EV studies. Plasma and serum are collected by different methods, and it is well-known that this difference results in the different profiles of proteins and nucleic acids between plasma and serum [15, 16]. Similarly, proteomic analysis of EV fractions isolated from plasma and from serum have suggested differences in protein profiles between these blood EVs [17, 18]. Mass spectroscopy (MS) analysis by Cao et al. reported that EV marker proteins were detected more abundantly in EV fractions from plasma than those from serum, suggesting that plasma EVs may be a more favorable source of blood EVs for MS-based proteomics [17]. Although these studies suggest that plasma and serum might not be equivalent sources of blood EVs, how EV profiles are different between plasma and serum, and the major factor leading to this difference has not been clarified to date.

To address this issue, in this study, we thoroughly characterized the profiles of blood EVs derived from mouse plasma and serum by nanoparticle tracking analysis (NTA) and proteomic analysis. We demonstrated that plasma and serum indeed show differences in the number of EV particles and their protein contents. Interestingly, we found that platelet-associated proteins are selectively enriched in the EV fractions derived from serum, both in mouse and human blood, which was further confirmed by a literature review of proteome data reported by others. These data indicate that EV fractions isolated from serum contain additional EVs that are released from platelets during the coagulation reactions occurring during serum preparation, resulting in the difference from plasma EV fractions. Our study provides experimental evidence that increased levels of platelet-derived particles in serum alter blood EV profiles, potentially affecting the results and the interpretation of studies on EVs, when using serum as a source of blood EVs.

## Materials and methods

### Blood samples

All mouse experiments were approved by the Animal Experiments Committee of Osaka University, and were performed in accordance with Osaka University Regulations on Animal Experiments. Eight-week old C57BL/6J male mice were purchased from Charles River Laboratories, Japan. All mice were acclimatized to the environment of the animal facility for 3 days, and were then subjected to blood sampling. The number of mice used in the study is indicated in the Figure legend. For preparation of plasma, whole blood (approximately 900 μL) was collected from each mouse through cardiac puncture without thoracotomy under isoflurane anesthesia and was immediately mixed with an anticoagulant solution (ethylenediaminetetraacetic acid [EDTA] disodium salt, sodium citrate, or heparin to a final concentration of approximately 4 mM, 0.38%, and 22 U/mL, respectively). Whole blood mixed with anticoagulants was kept on ice before centrifugation at 1,000 × g for 10 min at 4°C. As longer incubation of whole mouse blood with anticoagulants often increased the extent of hemolysis, centrifugation was performed within 10 min after the addition of anticoagulants to reduce the risk of hemolysis. After centrifugation, the transparent upper layer was collected as plasma, and stored at −80°C. For the preparation of serum, whole blood was similarly collected from mice through cardiac puncture without thoracotomy under isoflurane anesthesia, and was kept at room temperature for 30 min for the coagulation reaction. Blood samples were then centrifuged at 1,000 × g for 10 min at 4°C. After centrifugation, the transparent upper layer was collected as serum and stored at −80°C.

Human plasma and serum were prepared by Kohjin-Bio Co., Ltd. using the following protocol. Human subjects were all males, ranging from 21 to 39 years of age. Whole blood collection for plasma and serum was performed sequentially from the same individuals without any interval. For the preparation of plasma, whole blood was collected by venipuncture into collection tubes containing EDTA dipotassium salt. Immediately after collection, the blood samples were gently mixed with an anticoagulant by inverting 8 to 10 times, and were then centrifuged at 2,000 × g for 10 min at 4°C within 20 min after whole blood collection. For the preparation of serum, whole blood was collected by venipuncture into collection tubes, and then kept at room temperature for 30 min for the clotting reaction, followed by centrifugation at 2,000 × g for 10 min at 4°C. After centrifugation, the transparent upper layer was transferred to new tubes and stored at −80°C until use.

All blood samples from mice and humans were divided into small aliquots before freezing to avoid multiple freeze-thaw cycles. Blood samples in which the absorbance at 415 nm was greater than 0.2 after a 10-times dilution in phosphate-buffered saline (PBS) were considered to be hemolyzed and were not used for the analysis.

### Isolation of EVs from blood samples using ultracentrifugation

EV fractions were isolated from blood samples using a standard ultracentrifugation method. Briefly, 100 μL of plasma or serum was diluted with 4.9 mL of PBS, and centrifuged sequentially at 2,000 × g for 15 min and at 10,000 × g for 30 min to remove residual blood cells, including platelets, their debris, and large EVs. Supernatants were transferred to 13 × 51 mm Thinwall Polypropylene tubes (Beckman Coulter) and further centrifuged at 100,000 × g for 90 min using an Optima L-90K or XE-90 centrifuge (Beckman Coulter) together with an SW55Ti rotor. The pellet containing EVs was suspended in an appropriate buffer and used immediately for subsequent experiments, including transmission electron microscopy, nanoparticle tracking analysis, Western blotting, and proteomic analysis. All centrifugations were performed at 4°C.

### OptiPrep density-gradient centrifugation

OptiPrep (iodixanol, Axis-Shield) density gradient was prepared with 10% and 30% Optiprep solutions, 0.25 M sucrose, 1 mM EDTA, and 10 mM Tris (pH 7.4). The EV fractions in 0.25 M sucrose, 1 mM EDTA, 10 mM Tris (pH 7.4) (500 μL) were loaded on top of the OptiPrep density-gradient solution (11 mL) in 14 × 89 mm Thinwall Polypropylene tubes, and were subjected to ultracentrifugation at 200,000 × g using an SW41Ti rotor (Beckman Coulter) for 18 hours at 4°C. The subfractions (750 μL each) were then collected, and the density of each fraction was determined using a refractometer (Atago). Total proteins in each fraction were concentrated by trichloroacetic acid/acetone precipitation, and analyzed by Western blotting.

### Immunodepletion experiments

Immunodepletion of lipoprotein particles from the EV fractions was performed using antibodies against lipoproteins and Protein A-conjugated magnetic beads (Dynabeads, Invitrogen). Protein A beads were washed with 0.001% Tween 20 containing PBS supplemented with 0.1% bovine serum albumin (BSA), and incubated overnight with antibodies against Apolipoprotein A1 (ApoA1) (ab227455, rabbit polyclonal, Abcam), and Apolipoprotein B (ApoB) (ab20737, rabbit polyclonal, Abcam) at 4°C. The beads were then incubated overnight with the EV fractions at 4°C. After the incubation, the supernatants and the beads were separated by DynaMag-2 magnet, and analyzed by Western blotting and nanoparticle tracking analysis.

### Nanoparticle tracking analysis

Nanoparticle tracking analysis (NTA) was performed using NanoSight LM10. EV solutions were diluted with PBS to adjust particle concentrations to 30 to 80 particles/frame for NTA. The Brownian motions of nanoparticles in PBS were observed for 60 s at room temperature, and recorded five times for each measurement. Each sample was measured twice and averaged. All data were analyzed using NTA software v3.2, and the parameters were as follows: camera level, 16; detection threshold, 5.

### Transmission electron microscopy

Electron microscopic analysis of blood EVs was performed basically following a previously published method, with minor modifications [19]. Briefly, EV samples were mixed with equal volumes of 4% (wt/vol) paraformaldehyde (PFA)/PBS (final concentration, 2% PFA) and incubated overnight. The EV solutions were then loaded onto formvar-coated Cu grids (200 mesh, Nisshin EM) and incubated for 20 min. Excess solvent was removed with filter paper. The EV grids were washed with PBS twice, and then post-fixed with 1% glutaraldehyde for 5 min. After washes with water eight times, the EVs were negatively stained with uranyl acetate, and analyzed using the transmission electron microscope H-7650 (Hitachi).

Immunoelectron microscopy was performed as described previously [19]. Briefly, EV pellets were treated with PFA and loaded onto formvar-coated Cu grids (200 mesh), and incubated for 30 min. Excess solvent was removed with filter paper. The EV grids were washed three times with PBS and four times with 50 mM ammonium chloride. The grids were then blocked with PBS supplemented with 5% (w/v) BSA for 30 min, and incubated overnight with an anti-CD9 antibody (CBL162, mouse monoclonal, Merck Millipore) at 4°C. The grids were washed five times with PBS and incubated for 2 hours with a secondary antibody conjugated with 10-nm gold nanoparticles (G7777, Sigma-Aldrich). The immunolabeled grids were washed eight times with PBS and post-fixed with 1% glutaraldehyde for 5 min. After washes with water eight times, the EVs were negatively stained with uranyl acetate, and analyzed using the transmission electron microscope HT7700 (Hitachi). EV grids that were treated with the secondary antibody alone without the first antibody were prepared as controls.

### Mass spectroscopy and proteomic analysis

EV fractions were isolated from mouse blood plasma or serum as above. The proteins (7.6 μg per sample) in 50 mM Tris (pH 8.0), 2% sodium deoxycholate (SDC), 10 mM dithiothreitol with a protease inhibitor cocktail (Roche) were alkylated by iodoacetamide, reduced by tris(2-carboxyethyl)phosphine, and proteolyzed with a mixture of trypsin and lysyl endopeptidase (0.5 μg per sample) (Trypsin/Lys-C Mix, Promega). After removal of SDC according to the phase transfer surfactant protocol [20], the digested peptides were desalted, dried, and reconstituted in a loading buffer of 3% acetonitrile (ACN) plus 0.1% trifluoroacetic acid. The peptides were separated using an UltiMate 3000 Nano LC system equipped with an ESI-column (0.075 × 150 mm) (gradient: 5%–30% B in A [A = H_2_O containing 0.1% formic acid, B = ACN containing 0.1% formic acid] over 120 min; flow: 300 nL/min). The eluted peptides were detected by an in-line mass spectrometer (Q-Exactive, Thermo Scientific) operating in data-dependent analysis mode (35,000 high resolution, scan range 400–2,000 m/z, 5 × 10^5^ automatic gain control target, ~50 ms maximal ion time), followed by sequential isolation of the top 10 abundant ions for MS/MS analysis in the linear ion trap of low resolution (fragmentation by collision-activated dissociation with 32 normalized collision energy, 1 × 10^6^ automatic gain control target, ~30 ms maximal ion time, 2.0 m/z isolation window, and 20 s dynamic exclusion). MS/MS spectra were converted into a peak list using Mascot Distiller version 2.5 (Matrix Science) and searched against the UniProt protein database (release 2017_02: February 5, 2017, 51031 entries) using Mascot Server version 2.5 (Matrix Science). Search parameters included a mass tolerance of 10 ppm for precursor ions and 0.02 Da for MS/MS ions, with a maximum of one missed cleavage, maximum of three dynamic modification sites per peptide, carbamidomethylation of cysteine as a static modification, and oxidation of methionine and deamidation of asparagine and glutamine as dynamic modifications. The threshold score/expectation value for accepting individual spectra was based on Mascot ion score threshold (0.05) as the standard ion score threshold specifically calculated by Mascot for each database search. All matched MS/MS spectra were filtered by mass accuracy and matching scores to reduce the protein false discovery rate to about 1%. Gene ontology (GO) analysis, principal component analysis (PCA), and heat map analysis were conducted using DAVID Bioinformatics Resources 6.8 [21], MetaboAnalyst 4.0 [22] and R software with the heatmap.2 function, respectively.

### Western blotting

Equal amounts of total proteins of the EV fractions were separated using 5% to 20% gradient sodium dodecyl sulfate-polyacrylamide gel electrophoresis gels (ATTO), and transferred to PVDF membranes (Bio-Rad). The membranes were incubated overnight with the following primary antibodies at 4°C: anti-CD63 (10628D, mouse monoclonal, Thermo Fisher Scientific), anti-CD9 (CBL162, mouse monoclonal, Merck Millipore), anti-Hsc70 (ADI-SPA-819, rabbit polyclonal, Enzo Life Sciences), anti-GPIIb (LS-B13882, rabbit polyclonal, LifeSpan Biosciences), anti-GPIIIa (R30709, rabbit polyclonal, NSJ Bioreagents), anti-ApoB (ab20737, rabbit polyclonal, Abcam), and anti-ApoA1 (ab227455, rabbit polyclonal, Abcam) for both mouse and human samples, anti-PF4 (MAB595, rat monoclonal, R&D systems) for mouse samples, and anti-PF4 (ab129183, rabbit monoclonal, Abcam) for human samples. As secondary antibodies, horseradish peroxidase (HRP)-conjugated immunoglobulins were used. The HRP signal was visualized by ImmunoStar Zeta/LD (Wako), and captured using an Amersham Imager 600 system (Cytiva). Acquired images were analyzed using ImageJ software (NIH).

### Statistical analysis

Statistical analyses were performed to assess differences among groups by one-way analysis of variance (ANOVA) followed by the Tukey multiple comparison test for the NTA results in Fig 2, and to assess differences between human plasma and serum EVs by the Student *t*-test (paired) in Fig 5 and S5 Fig. For other comparisons, the Student *t*-test (unpaired) was performed. Data are expressed as the mean ± standard error of the mean (SEM). For all statistical analyses, GraphPad Prism software (GraphPad Software Inc.) was used. A *P*-value of less than 0.05 was considered to indicate a statistically significant difference between groups. For calculation of the effect size, the power analysis software G*Power 3.1 [23] was used.

## Results

### Serum sEV fractions contain more EV particles than plasma sEV fractions in mouse blood

**Fig 1:**
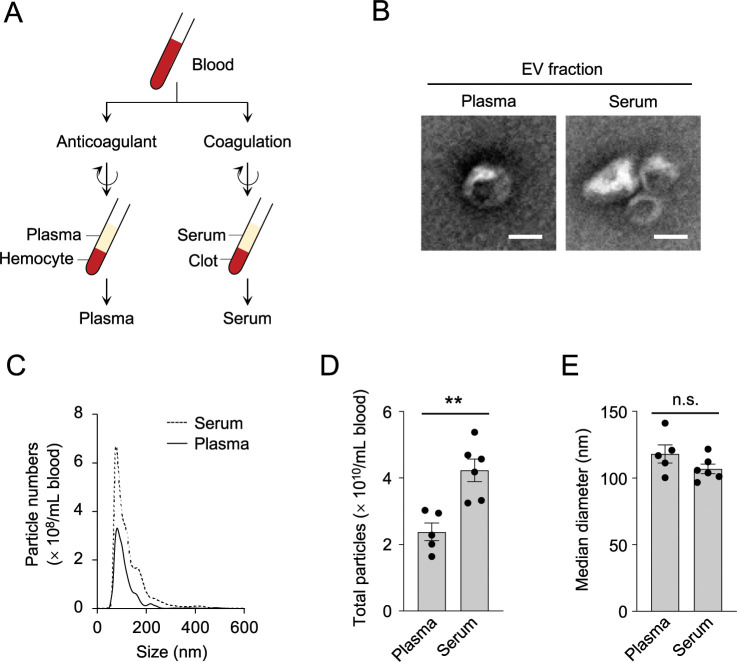
Serum sEV fractions contain more EV particles than plasma sEV fractions in mouse blood. (A) Schematic representation of the preparation methods of plasma and serum. Plasma was separated from anticoagulant-treated blood by removing hemocytes and other precipitates by centrifugation. Serum was separated from coagulated blood by removing blood clots by centrifugation. (B) Transmission electron micrographs of particles in the sEV fractions derived from plasma and serum. Scale bars, 100 nm. (C-E) NTA of the sEV fractions derived from plasma and serum. A plot showing the size distribution of the EV particles (C) and bar graphs showing the numbers (D) and median diameters (E) of the particles in the sEV fractions isolated from plasma and serum. Data are shown as the average ± SEM (plasma, n = 5; serum, n = 6). **P < 0.01, Student *t*-test. n.s., not significant. Experiments were performed independently at least three times, and representative data are shown. In C and D, particle numbers are normalized to original blood volumes.

To analyze whether plasma and serum are comparable sources of blood EVs, we prepared plasma and serum from male C57BL/6J mice (Fig 1A), and isolated EV-containing fractions by the conventional ultracentrifugation method. As we used ultracentrifugation for EV purification, the predominant fraction of EVs analyzed in this study were comprised mostly of relatively small particles, *i*.*e*., small EVs (sEVs), rather than large EVs. For the preparation of plasma, EDTA was used as an anticoagulant unless otherwise noted. Transmission electron microscopic observation showed that both sEV fractions isolated from mouse plasma and serum contain cup-shaped vesicles with a diameter of approximately 100 nm (Fig 1B), which were immunopositive for CD9, which is an EV marker (S1A Fig). These results are in good agreement with the morphological nature of sEVs reported in the literature [24]. NTA using NanoSight LM10 unexpectedly demonstrated a significant difference in particle numbers between plasma and serum (Fig 1C). The particle number (± standard deviation) for plasma EVs was 2.38 ± 0.60 (× 10^10^/mL blood, n = 5), whereas that for serum EVs was 4.23 ± 0.83 (× 10^10^/mL blood, n = 6), which was calculated to be a 78% increase in the number of particles in the sEV fractions derived from serum, compared with those from plasma (*P* = 0.0024, Student *t*-test; effect size, 2.56) (Fig 1D). On the other hand, the diameters of the particles in the sEV fractions were distributed comparably within the range of 50 nm to 300 nm (Fig 1C); the median diameter was 118.1 ± 15.1 nm for plasma EVs *vs*. 106.9 ± 8.9 nm for serum EVs, which was not statistical different (*P* = 0.16, Student *t*-test; effect size, 0.90) (Fig 1E). EV fractions after ultracentrifugation are known to contain lipoproteins, protein aggregates, and other non-EV particles [25, 26], which may affect the correct estimation of the particle count in the NTA. To validate the above findings using blood EVs with fewer contaminants, we tried to reduce the contamination of lipoprotein particles in the sEV fractions by conducting immunodepletion using antibodies against lipoproteins. As show in S1B Fig, ApoB and ApoA1, which are major lipoproteins in blood, were reduced in the supernatant fractions after immunodepletion with these lipoprotein antibodies, but were significantly accumulated on the beads, whereas CD63 and Hsc70 were unchanged, indicating the successful reduction of lipoprotein levels by immunodepletion. NTA of the supernatants after immunodepletion demonstrated that the sEV fractions derived from mouse serum have a consistently larger number of particles than those from plasma (1.49 ± 0.21 [× 10^9^/mL] for plasma EVs vs. 5.54 ± 0.85 [× 10^9^/mL] for serum EVs; *P* = 0.02, Student *t*-test; effect size, 6.53), with no difference in particle diameter (110.2 ± 9.1 nm for plasma EVs vs. 109.5 ± 3.6 nm for serum EVs; *P* = 0.87, Student *t*-test; effect size, 0.10) (plasma, n = 5; serum, n = 6) (S1C Fig). These results suggest that EVs from both blood sources show no apparent difference in size distribution, but the serum contains more EVs than plasma.

**Fig 2:**
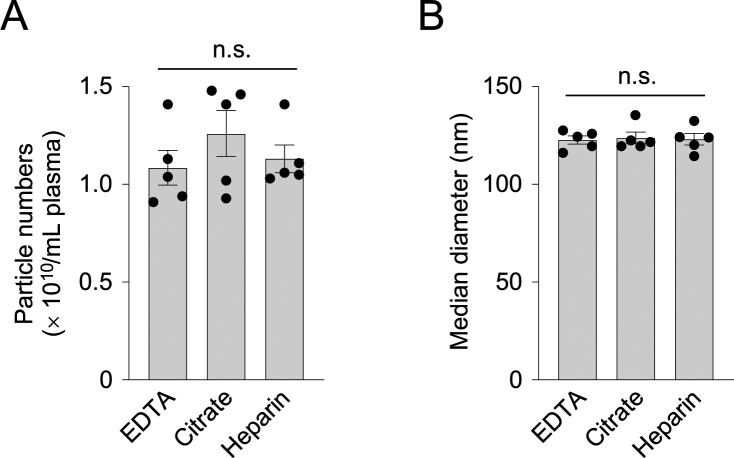
Anticoagulants have no detectable effects on numbers and diameters of EVs in plasma. (A-B) Bar graphs showing numbers (A) and median diameters (B) of the EVs that were isolated from plasma prepared using EDTA, citrate, and heparin. Data are shown as the average ± SEM (n = 5). Statistical analysis was performed by one-way ANOVA followed by the Tukey multiple comparisons test. n.s., not significant.

Several anticoagulants are routinely used for the preparation of plasma from whole blood. Among them, EDTA, citrate, and heparin are three major anticoagulants. EDTA and citrate inhibit blood clotting by chelating the calcium ions that are necessary for the coagulation cascade. Heparin binds to and activates antithrombin, leading to the inactivation of proteases involved in the coagulation cascade, such as thrombin and factor Xa. To investigate whether anticoagulants affect the number and size distribution of the resultant plasma EVs, we prepared mouse plasma using three different anticoagulants, and isolated the sEV fractions by ultracentrifugation, which were subjected to the NTA experiments as above (n = 5). NTA of these sEV fractions showed no detectable difference in the number and diameter of the plasma EVs (particle number: 1.09 ± 0.20 [× 10^10^/mL] for EDTA, 1.26 ± 0.26 [× 10^10^/mL] for citrate, 1.13 ± 0.16 [× 10^10^/mL] for heparin; *P* = 0.43, one-way ANOVA; effect size, 0.35) (diameter: 122.8 ± 4.7 nm for EDTA, 123.8 ± 6.6 nm for citrate, 123.1 ± 6.6 nm for heparin; *P* = 0.96, one-way ANOVA; effect size, 0.07) (Fig 2), suggesting that these anticoagulants do not affect the yield and size of the EV particles in plasma.

### Serum sEV fractions contain a larger amount of platelet-associated proteins than plasma sEV fractions from mouse blood

**Fig 3:**
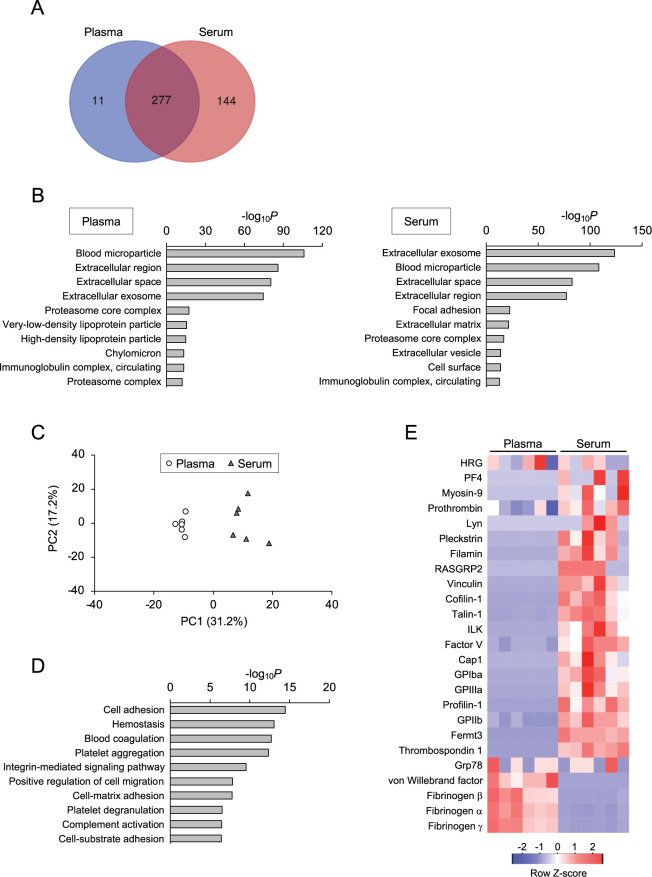
Proteomic analysis demonstrates substantial enrichment of platelet-associated proteins in serum EVs. (A) Venn diagram of the proteins identified in the sEV fractions derived from plasma and serum (n = 6) by LC-MS/MS analysis. (B) GO cellular component of the proteins identified in plasma EVs and serum EVs. *P*-values from the modified Fisher’s exact test are shown as bar graphs. (C) Principal component analysis of the dataset of proteins identified in plasma EVs and serum EVs. (D) GO biological process of the proteins that are upregulated or exclusively detected in serum EVs. (E) Heat map analysis of proteins with platelet-associated GO terms that were identified in our LC-MS/MS analysis.

To analyze whether the EV contents are different between plasma and serum, we performed proteomic analysis of sEV fractions that were isolated by ultracentrifugation from mouse blood (n = 6). Semiquantitative LC-MS/MS analysis identified 288 and 421 proteins in the plasma and serum sEV fractions, respectively, and 432 proteins in total. Among these, 277 proteins were commonly detected in both sEV fractions, and 11 and 144 proteins were detected only in the plasma and serum sEV fractions, respectively (Fig 3A, S1 Table). GO analysis demonstrated substantial enrichment of EV-associated proteins in both serum and plasma, as the GO terms (cellular component, CC) associated with EVs, such as ‘extracellular exosome,’ ‘blood microparticle,’ and ‘extracellular region/space,’ were commonly detected as top hits (Fig 3B). GO analysis of the other groupings apparently showed similar GO profiles between the plasma and serum sEV fractions; in GO biological process (BP), the term ‘complement activation, classical pathway’ was the most enriched, followed by ‘innate immune response,’ ‘hemostasis,’ and ‘blood coagulation’ (S2A Fig), and in GO molecular function (MF), the term ‘antigen binding’ was the most enriched, followed by ‘serine-type endopeptidase inhibitor activity,’ ‘peptidase inhibitor activity,’ and ‘threonine-type endopeptidase activity’ (S2B Fig).

To analyze the protein profiles in more detail, we then performed principal component (PC) analysis of the LC-MS/MS datasets, which consist of a list of identified proteins and the number of the corresponding peptide fragments detected by LC-MS/MS analysis; the latter of which can be considered to reflect the relative amount of each protein. PC analysis showed that plasma EV and serum EV data are plotted as different clusters in the PC space without any overlap (Fig 3C), suggesting that protein contents are substantially different between the plasma and serum sEV fractions.

To analyze how the protein contents are different between these sEV fractions, we focused on a group of proteins that are increased in the serum EVs. Among the 432 proteins that were identified by LC-MS/MS analysis, 144 proteins were exclusively detected in serum EVs, and 28 proteins were commonly detected in both types of EVs, but were upregulated by more than 2-fold in the serum EVs compared with the plasma EVs (*P* < 0.05) (S2 Table). GO analysis focusing on these 172 proteins that were increased in serum EVs clearly showed the substantial enrichment of proteins that are involved in the blood coagulation cascade, as the GO terms including ‘hemostasis,’ ‘blood coagulation,’ and ‘complement activation’ were listed as the top 10 terms (Fig 3D, S3 Fig). In addition, proteins associated with platelets were also suggested to be enriched, as the GO terms ‘platelet aggregation,’ and ‘platelet degranulation’ (BP) and ‘blood microparticle’ (CC) were detected (Fig 3D, S3 Fig). Because platelets produce EVs called microparticles or microvesicles, and secrete them into the blood, particularly when they are activated during blood coagulation [27, 28], serum might contain these extracellular particles released from platelets more abundantly than plasma. Consistently, proteins that are specifically expressed in platelets, such as platelet membrane glycoproteins IIb/IIIa (GPIIb/IIIa) and Ib-IX-V, were all found to be increased or exclusively detected in the serum EVs (Table 1). Integrin alpha-2/beta-1 (a collagen receptor) and platelet factor 4 (PF4, a protein stored in α granules, a platelet-specific organelle) are dominantly expressed in platelets and were also increased in the serum EVs. Furthermore, proteins that bind to platelet-specific glycoproteins, such as talin-1 and fermitin family homolog 2/3, were upregulated in the serum EVs. To analyze the general tendency of the selective enrichment of platelet-associated proteins in serum sEV fractions, we analyzed all the platelet-associated proteins detected in the present study. Among the 432 proteins that were identified in our LC-MS/MS analysis, 25 proteins have GO terms that are apparently associated with platelets, including ‘platelet degranulation,’ ‘platelet activation,’ and ‘platelet aggregation’ (BP), and ‘platelet alpha granule’ (CC) (Fig 3E). Heat map analysis focusing on these platelet-associated proteins clearly showed the overall tendency of upregulation of these proteins in serum EVs, compared with plasma EVs (Fig 3E); the only exceptions were fibrinogens (α, β, and γ) and von Willebrand factor, which appeared to be increased in plasma EVs, possibly because these proteins coprecipitate with other clotting factors during coagulation, and are eventually removed from serum. These findings indicate that the levels of proteins that are specific to or closely associated with platelets are selectively increased in serum EVs.

**Table 1:** Platelet-associated proteins detected in serum EVs.

Protein	Gene name	Alternative name(s)	Fold change (serum/plasma)
Glycoprotein IIb/IIIa			
Integrin alpha-IIb	Itga2b	GPIIb, CD41	22
Integrin beta-3	Itgb3	GPIIIa, CD61	Only in serum
Glycoprotein Ib-IX-V receptor complex			
Platelet glycoprotein Ib alpha chain	Gp1ba	GPIbα, CD42b	Only in serum
Glycoprotein Ib, beta polypeptide	Gp1bb	GPIbβ, CD42c	Only in serum
Platelet glycoprotein IX	Gp9	GPIX, CD42a	Only in serum
Platelet glycoprotein V	Gp5	GPV, CD42d	Only in serum
α granule proteins			
Coagulation factor V	F5	−	13.75
Platelet factor 4	Pf4	−	Only in serum
Proteins expressed in platelets			
Integrin alpha-2	Itga2	GPIa, CD49b	Only in serum
Integrin beta-1	Itgb1	GPIIa, CD29	Only in serum
Platelet glycoprotein 4	Cd36	GPIIIb, GPIV	Only in serum
Pleckstrin	Plek	−	Only in serum
Integrin αIIbβ3 binding proteins			
Talin-1	Tln1	−	104.33
Filamin, alpha	Flna	−	Only in serum
Leukocyte surface antigen CD47	Cd47	−	2.25
4F2 cell-surface antigen heavy chain	Slc3a2	a part of CD98/LAT1	Only in serum
Fermitin family homolog 3	Fermt3	Kindlin-3	Only in serum
Fermitin family homolog 2	Fermt2	Kindlin-2	Only in serum
Integrin-linked protein kinase	Ilk	−	Only in serum
Tyrosine-protein kinase Lyn	Lyn	−	Only in serum
Glycoprotein Ib-IX-V binding proteins			
14-3-3 protein zeta/delta	Ywhaz	−	Only in serum

**Fig 4:**
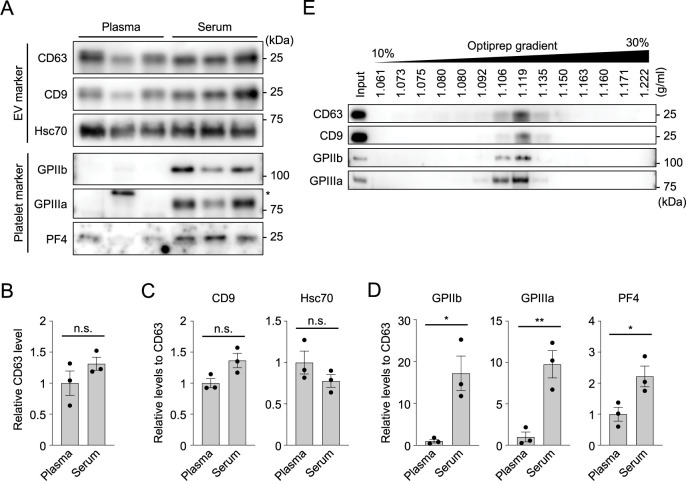
Platelet-specific proteins are abundantly detected in sEV fractions derived from serum of mouse blood. (A-D) Western blot analysis of the sEV fractions derived from plasma and serum using antibodies against EV marker proteins and platelet-specific proteins (A) and their densitometric analyses (B-D). Equal amounts of proteins were loaded onto each lane and analyzed (n = 3). The level of CD63 (B) and the levels of EV markers CD9 and Hsc70 relative to CD63 (C) showed no statistical difference between plasma and serum EVs. In contrast, platelet-specific GPIIb, GPIIIa, and PF4 were significantly increased in the serum sEV fractions (D). The asterisk in (A) indicates a nonspecific signal. (E) Optiprep density-gradient centrifugation analysis of the serum sEV fraction with EV marker proteins and platelet-specific proteins. GPIIb and GPIIIa were both detected in the fractions at the same density as the sEV, and cofractionated with EV markers. Data are shown as the average ± SEM. *P < 0.05, **P < 0.01, Student *t*-test. n.s., not significant.

To validate the results from the proteomic analysis, we prepared the sEV fractions from mouse serum and plasma by ultracentrifugation, and analyzed the levels of platelet-associated proteins by Western blot analysis (n = 3) (Fig 4A). The levels of EV marker proteins, such as CD63, CD9, and Hsc70 were comparable between plasma and serum EVs (*P* = 0.23, 0.06, and 0.23, respectively, Student *t*-test; effect size, 1.14, 2.19, and 1.15, respectively) (Fig 4A–4C). In contrast, the levels of platelet-associated proteins, such as GPIIb, GPIIIa, and PF4 were significantly increased in the serum sEV fractions (*P* = 0.017, 0.007, and 0.038, respectively, Student *t*-test; effect size, 3.21, 4.10, and 2.48, respectively) (Fig 4D), which is consistent with the results of our LC-MS/MS measurements. Density-gradient centrifugation analysis of the serum sEV fractions using OptiPrep demonstrated cofractionation of GPIIb and GPIIIa with the EV markers CD63 and CD9, at a density of 1.10–1.13 g/mL (Fig 4E), consistent with the density of sEVs reported in the literature [29]. In contrast, the plasma sEV fractions did not show clear signals of platelet-associated GPIIIa in any fractions separated by density-gradient centrifugation (S4 Fig). These results suggest that platelet-associated proteins that are increased in serum EVs are indeed released via EVs.

### Platelet-associated proteins are generally enriched in serum sEV fractions of human blood

**Fig 5:**
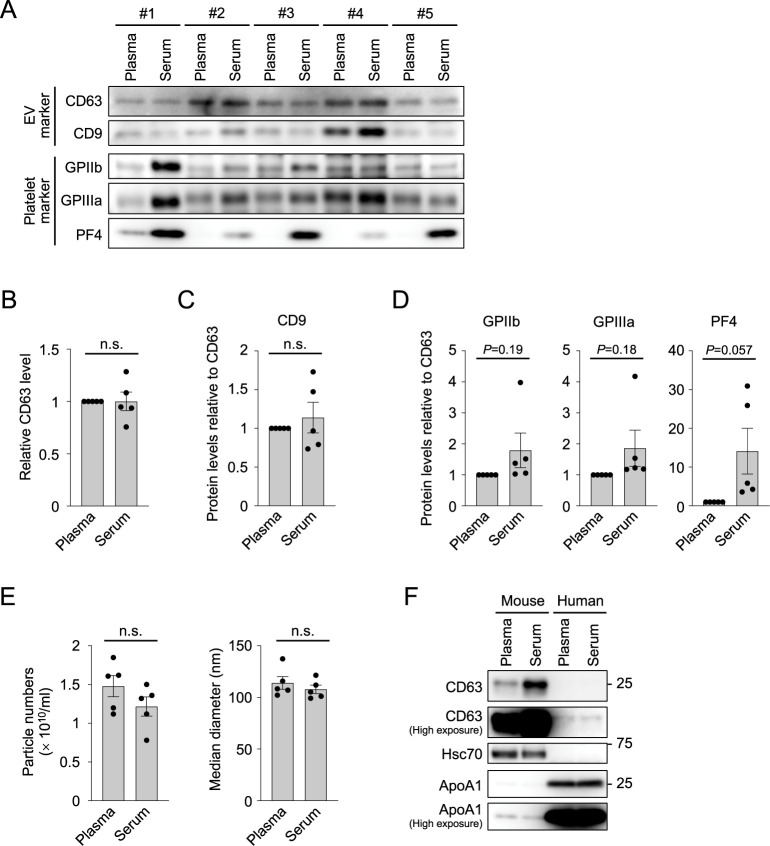
Serum EVs contain high levels of platelet proteins in human blood. (A) Western blot analysis of the sEV fractions derived from plasma and serum of 5 individuals, using antibodies against EV markers and platelet-specific proteins. Equal amounts of proteins were loaded onto each lane and analyzed. (B) Bar graph showing relative protein levels of CD63 that were calculated from the band intensity of the Western blot image in (A). (C-D) Bar graphs showing the levels of the EV marker CD9 (C), and platelet-specific markers GPIIb, GPIIIa and PF4 (D), relative to that of CD63. Whereas the level of CD9 was comparable between the sEV fractions from plasma and serum, all the platelet markers showed a clear tendency of increased levels in serum EVs. (E) NTA results showing the numbers (left) and median diameters (right) of the particles in the sEV fractions derived from human plasma and serum. (F) Comparison of the sEV fractions derived from mouse and human blood by Western blot analysis using antibodies against EV markers and lipoprotein ApoA1. The sEV fractions isolated from human blood contain very low levels of the EV markers CD63 and Hsc70, but a high level of ApoA1, implying a large difference in particle population between human and mouse blood. Data are shown as the average ± SEM. Student *t*-test. n.s., not significant.

We next analyzed whether serum EVs isolated from human blood also contain higher levels of platelet-associated proteins than plasma EVs, as observed for mouse blood. Human blood was collected from five healthy individuals. sEV fractions were then isolated from human plasma and serum by the ultracentrifugation method, and subjected to Western blot analysis (Fig 5A). The EV marker proteins CD63 and CD9 were comparably present in both plasma and serum sEV fractions in each blood sample (*P* = 0.98 and 0.52, respectively; Student *t*-test) (Fig 5A–5C). In contrast, the levels of platelet-associated proteins, including GPIIb, GPIIIa, and PF4, showed increased tendencies in all serum EV samples compared with plasma EV samples (Fig 5A). Although densitometric analysis of the Western blot images did not show statistically significant differences in the levels of GPIIb, GPIIIa, and PF4 between plasma and serum (*P* = 0.19, 0.18, and 0.057, respectively; Student *t*-test) owing to large variances between individuals (Fig 5D), a clear trend of a selective increase in platelet-associated proteins in serum is in good agreement with the results observed in mouse blood experiments (Fig 4). These results suggest that platelet proteins are also increased in the sEV fractions from human serum.

We also analyzed whether human blood EVs show differences in number and size distributions between plasma and serum. NTA of the sEV fractions, which were isolated from 5 individuals by ultracentrifugation, demonstrated no detectable difference in the numbers and median diameters of the EV particles between plasma and serum (particle number: 1.48 ± 0.31 [× 10^10^/mL] for plasma EVs and 1.22 ± 0.28 [× 10^10^/mL] for serum EVs; *P* = 0.05, Student *t*-test; effect size, 1.28) (diameter: 114.1 ± 13.9 nm for plasma EVs, 107.8 ± 9.0 nm for serum EVs; *P* = 0.06, Student *t*-test; effect size, 1.13) (Fig 5E), which is inconsistent with the results observed for mouse blood; the particle number was higher in the serum sEV fractions than in the plasma sEV fractions in mouse blood (Fig 1). This discrepancy might be attributed to the different population of extracellular particles included in the sEV fractions from mouse and human blood. A comparison of mouse and human sEV fractions, which were both isolated by ultracentrifugation, using Western blot analysis demonstrated that sEV fractions from mouse blood contain high levels of the EV marker proteins CD63 and Hsc70, whereas those from human blood contain a high level of lipoprotein ApoA1, a marker of high-density lipoprotein (HDL) (Fig 5F). This result implies that human blood sEV fractions contain a much larger amount of lipoprotein-positive particles than mouse blood sEV fractions. Because not only EVs but also non-EV particles, including lipoprotein particles, are counted in the NTA, a large amount of non-EV particles in the EV fractions may interfere with the results of particle number analyses of EVs in human blood. Similarly, in S1B and S1C Fig, to assess the particle number of EVs under conditions with less contaminants, we performed immunodepletion of lipoproteins for human samples. Western blot analysis demonstrated that, although substantial accumulation of ApoB and ApoA1 on the beads after immunodepletion with antibodies against these lipoproteins was observed, in the supernatant, a slight decrease in the protein level of ApoA1 but not ApoB was detected (S5A Fig). NTA of the supernatants after immunodepletion showed a tendency of more EVs in the sEV fractions from serum than those from plasma, but the difference was not statistically significant (particle number: 7.00 ± 2.01 [× 10^9^/mL] for plasma EVs and 7.68 ± 1.22 [× 10^9^/mL] for serum EVs; *P* = 0.27, Student *t*-test; effect size, 0.57) (diameter: 117.7 ± 13.3 nm for plasma EVs, 113.4 ± 7.5 nm for serum EVs; *P* = 0.30, Student *t*-test; effect size, 0.53) (S5B Fig). These results suggest that the removal of lipoproteins by immunodepletion can be partly achieved under the tested condition, but is apparently insufficient, particularly for human EV samples containing large amounts of lipoproteins. Further optimization of the experimental conditions and purification methods to remove non-EV particles, such as lipoproteins, is required for the precise analysis of human blood EVs.

**Fig 6:**
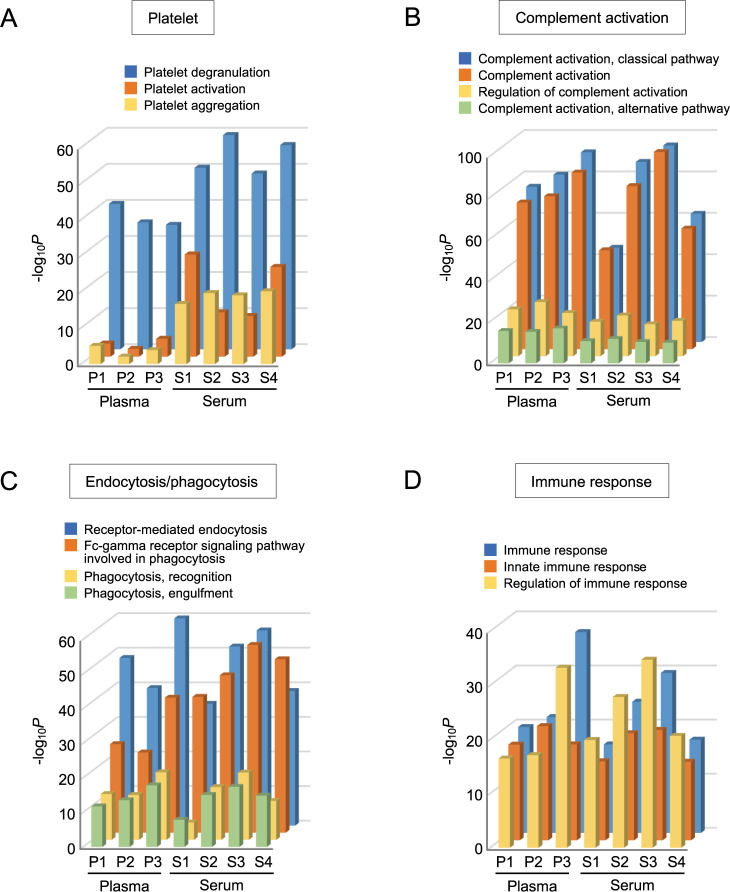
Platelet-associated proteins are generally enriched in serum EVs. (A-D) Bar graphs showing modified Fisher’s exact *P*-values of the GO biological process terms associated with platelets (A), complement activation (B), endocytosis/phagocytosis (C), and immune response (D), which were calculated from the proteomic data of plasma EVs (P1, Zheng et al.; P2, Yentrapalli et al.; P3, Bezdan et al.), and serum EVs (S1, Zhong et al.; S2, An et al.; S3, Ding et al.; S4, Luo et al.) reported from other groups. The GO terms associated with platelets were significantly enriched in serum EVs compared with plasma EVs, whereas the other GO terms showed almost comparable enrichment among the studies analyzed.

We then asked whether the increased levels of platelet-associated proteins in serum EV fractions generally affect the content profiling of human blood EVs. To test this possibility, we searched recently published studies reporting proteomic analyses of sEV fractions isolated by ultracentrifugation of either human plasma [30–32] or serum [33–36] (S3 Table), and analyzed these studies focusing on the enrichment of platelet-associated proteins. GO analysis demonstrated that the GO biological process terms associated with platelets were generally more frequent in studies using human serum EVs, compared with those using human plasma EVs (Fig 6A, S6 and S7 Figs). In contrast, other top GO terms associated with complement activation, endocytosis/phagocytosis, and the immune response were observed at comparable frequencies in studies of both plasma and serum EVs (Fig 6B–6D, S6 and S7 Figs). These results confirm that the selective enrichment of proteins associated with platelets is a commonly shared aspect of serum EV studies, and affects the proteome profiles of blood EVs.

## Discussion

Multiple factors are known or suspected to affect blood EV profiles, including methods of blood collection, hemolysis, the choice of serum/plasma, anticoagulants, freeze/thaw cycles, storage duration/temperature, as well as EV isolation methods [2]. Although the experimental results and their interpretation highly depend on these multiple factors, whether, how, and to what extent these factors affect EV profiles and subsequent analysis results have not been extensively investigated. Among these factors, the issue of the difference between plasma and serum as a source of blood EVs is fundamental, and often encountered in clinical studies using stored patient samples, in which the use of either plasma or serum cannot be chosen owing to limited availability. In the present study, to analyze the effects of the choice of either plasma or serum on EV profiles, we performed a comparison of the sEV fractions of plasma and serum, which were isolated by a standard ultracentrifugation method. Using mouse blood prepared under the controlled experimental conditions, we demonstrated that plasma and serum are qualitatively and quantitatively different as sources of blood EVs. We further showed that platelet-associated proteins that are released via EVs are generally enriched in serum sEV fractions, resulting in different proteome profiles of EVs between plasma and serum, both in mouse and human blood. These results indicate that increased levels of platelet-derived EVs in serum potentially affect the content profiles of isolated blood EV fractions when using serum as a source of blood EVs.

Our proteomic analysis clarified that sEV fractions derived from serum contain a large amount of proteins that are associated with platelets, including the platelet-specific receptor complexes GPIIb/IIIa and GPIb-IX-V, and α-granular proteins, together with proteins that are dominantly expressed in platelets (Figs 3 and 4, Table 1). This finding is in good agreement with previous studies suggesting the increased levels of platelet-associated membrane fragments or EVs in serum [18, 37]. It is known that platelets release not only a variety of growth factors, chemokines, and other bioactive mediators, but also EVs, such as platelet-derived microparticles and exosomes, particularly when they are activated [38]. Because serum collection requires blood coagulation, which activates EV release from platelets, it is supposed that serum contains, to some extent, additional EV particles that are produced by activated platelets. Considering our proteomic data showing the substantial enrichment of platelet-associated proteins in serum sEV fractions, serum EVs are highly contaminated by platelet-derived EVs. In support of this idea, our NTA using mouse blood unexpectedly demonstrated that the sEV fractions from serum contain a larger number of EV particles than those from plasma (Fig 1). This result implies that a considerable proportion of EV particles detected in serum might not actually be present in circulating blood, but are produced during serum preparation; however, the possibility that a proportion of blood EVs might be lost during plasma preparation or during EV isolation from plasma cannot be excluded, and should be analyzed in a future study. Collectively, we conclude that the contamination of serum with platelet-derived EVs that are produced during coagulation is a major factor leading to the difference in EVs derived from plasma and serum, resulting in alterations of the content profiles of serum EVs.

It has been reported that calcium chelators, such as EDTA and citrate, but not heparin, promote the association of EVs and platelets, and lower the apparent count of EV particles in plasma [39]. Palviainen et al. also demonstrated that the use of EDTA, citrate, and acid citrate dextrose (ACD) results in differences in particle number and protein profiles of plasma EVs [18]. In the present study, using blood samples derived from mice, however, we could not detect any difference in the numbers and diameters of EVs in plasma collected using EDTA, citrate, and heparin (Fig 2), and therefore, the use of anticoagulants may have little effect on EV profiles in plasma under the present condition. Multiple factors, such as sample sources (human *vs*. mouse), EV isolation methods (ultracentrifugation with various modifications), and analytical methods (flowcytometry *vs*. NTA) may affect the yield, purity, and cargo profiles of isolated EVs between studies, and therefore, further validation under controlled experimental conditions are needed [40].

In contrast to the mouse blood experiments, serum EVs isolated from human blood did not show a higher particle number than plasma EVs by NTA (Fig 5E). Our Western blot analyses suggest that the sEV fractions from human blood contain a much higher number of lipoprotein particles than those from mouse blood (Fig 5F). Indeed, a previous study showed that lipoprotein particles are coisolated with EVs when using ultracentrifugation, and the ratio of EV:HDL particles was estimated to be about 1:100 in human blood EV fractions [25]. Because EVs and other lipoprotein particles are both detected by NTA but cannot be distinguished, a large population of lipoprotein particles likely interferes with the particle measurement of human blood EVs. Further purification methods that result in minimal contamination of lipoprotein particles are necessary to enable the precise characterization of human blood-derived EVs [25, 26, 41].

Platelet EVs contain cytokines, eicosanoids, coagulation factors, and RNA species, which are involved in coagulation, inflammation, and the modulation of other cellular activities via their interaction with other cells [38]. Because our data demonstrated that serum contains additional EVs that are released from platelets, there is no doubt that the components of platelet EVs, including proteins, nucleic acids, lipids, and other metabolites, substantially affect the content profiles of serum EV fractions. The selective enrichment of platelet-associated proteins is commonly observed in proteomic analyses of human serum EVs, and interferes with the precise characterization of the content profiles of blood EVs (Fig 6). Nonetheless, our OptiPrep density gradient centrifugation analysis showed that platelet marker proteins, such as GPIIb/IIIa, are cofractionated in exactly the same density fraction as EV markers (Fig 4E), and therefore, selective exclusion of platelet EVs from the EV fractions isolated from serum would be quite challenging using the present purification methods. The development of methodologies that enable minimization of the contamination of serum with platelet EVs, including novel separation methods and/or data normalization, would be very useful, particularly for the analysis of clinically stored serum samples with limited availability.

## Supporting information

S1 Fig Immuno-EM and immunodepletion of sEV fractions derived from mouse blood. (A) Immuno-EM images of the EVs derived from mouse serum. The EVs were stained with a CD9 antibody and a secondary antibody conjugated with 10-nm gold nanoparticles. For the control experiment, the EVs were treated with only the secondary antibody. Scale bar, 50 nm. (B) Western blot analysis of the supernatant and beads fractions after immunodepletion of apolipoproteins from mouse plasma EVs using antibodies against ApoB and ApoA1. After immunodepletion using antibodies against ApoB and ApoA1, the signals of both apolipoproteins were significantly decreased in the supernatant, whereas signals of CD63 and Hsc70 remained unchanged. Correspondingly, strong signals of apolipoproteins were observed in the beads fraction. These results suggest that particles containing apolipoproteins ApoB and ApoA1 are partly, but not completely, removed from the sEV fractions by immunodepletion. IgG, immunoglobulin G. (C) NTA results of the blood sEV fractions from mice after immunodepletion using antibodies against ApoB and ApoA1. Data are shown as the average ± SEM (plasma, n = 5; serum, n = 6). ****P < 0.0001, Student *t*-test; n.s., not significant.
(EPS)

S2 Fig GO analysis of proteins identified in plasma EVs and serum EVs. (A-B) GO analysis (A, biological process; B, molecular function) of the proteins identified in the plasma EVs (top) and the serum EVs (bottom). Modified Fisher’s exact *P*-values of the top 10 GO terms are shown in the bar graphs.
(EPS)

S3 Fig GO analysis of proteins that were increased in serum EVs. (A-B) GO analysis (A, cellular component; B, molecular function) of the proteins that were increased in the serum EVs. Modified Fisher’s exact *P*-values of the top 10 GO terms are shown in the bar graphs.
(EPS)

S4 Fig Density-gradient centrifugation analysis of proteins included in the plasma sEV fraction. The sEV fractions were prepared by ultracentrifugation from mouse plasma and further fractionated by Optiprep density-gradient centrifugation, which were subjected to Western blotting using antibodies against CD63, CD9 and GPIIIa.
(EPS)

S5 Fig Immunodepletion of the sEV fractions derived from human blood. (A) Western blot analysis of the supernatant and beads fractions after immunodepletion of apolipoproteins from human plasma EVs using antibodies against ApoB and ApoA1. Although significant accumulation of ApoB and ApoA1 on the beads after immunodepletion with antibodies against these lipoproteins was observed, only a slight decrease in the protein level of ApoA1, and no decrease in ApoB was observed. (B) NTA results of human blood sEV fractions after immunodepletion using antibodies against ApoB and ApoA1. Data are shown as the average ± SEM (plasma, n = 5; serum, n = 5). Student *t*-test; n.s., not significant.
(EPS)

S6 Fig Modified Fisher’s exact *P*-values of the GO biological process terms that were calculated with published proteomic data of plasma EVs. Modified Fisher’s exact *P*-values of the GO biological process terms that were calculated with proteomic data of plasma EVs reported by Zheng et al. (P1), Yentrapalli et al. (P2), and Bezdan et al. (P3). The GO terms associated with platelets, complement activation, endocytosis/phagocytosis, and immune response are indicated in red, blue, green, and yellow, respectively.
(EPS)

S7 Fig Modified Fisher’s exact *P*-values of the GO biological process terms that were calculated with published proteomic data of serum EVs. Modified Fisher’s exact *P*-values of the GO biological process terms that were calculated with proteomic data of serum EVs reported by Zhong et al. (S1), An et al. (S2), Ding et al. (S3), and Luo et al. (S4). The GO terms associated with platelets, complement activation, endocytosis/phagocytosis, and immune response are indicated in red, blue, green, and yellow, respectively.
(EPS)

S1 Raw images Original uncropped images of the Western blots. (ZIP)

S1 Table List of proteins detected in mouse blood EVs. (XLSX)

S2 Table Proteins upregulated in mouse serum EVs. (XLSX)

S3 Table List of proteins detected in human blood EVs reported in other studies. (XLSX)

S4 Table Raw data of MS analysis of mouse blood EVs. (XLSX)
